# Dynamic brain network modulation by paced breathing and breath-holding: an EEG-based functional connectivity study

**DOI:** 10.3389/fphys.2025.1722715

**Published:** 2025-12-04

**Authors:** Kai Chen, Yanfang Zhang, Jiahao Cheng, Bharat B. Biswal, Tao Zhang, Junqiu Zhou

**Affiliations:** 1 Mental Health Education Center and School of Big Health Management, Xihua University, Chengdu, China; 2 Department of Neurology, Baiyun District People’s Hospital of Guangzhou, Guangzhou, Guangdong, China; 3 Department of Ultrasound Medicine, Baiyun District People’s Hospital of Guangzhou, Guangzhou, Guangdong, China; 4 The Clinical Hospital of Chengdu Brain Science Institute, MOE Key Laboratory for Neuroinformation, Center for Information in Medicine, School of Life Science and Technology, University of Electronic Science and Technology of China, Chengdu, China

**Keywords:** breathing, brain network, forced vital capacity, functional connectivity, EEG

## Abstract

**Introduction:**

Growing evidence shows that voluntary breathing maneuvers modulate cortical oscillations, yet the precise frequency-specific signatures of functional connectivity (FC) remain unclear.

**Methods:**

This study investigated the impact of different respiratory conditions on brain FC using EEG recordings. Three respiratory conditions were collected and analyzed: self-paced breathing (SB), breath-holding (BH), and computer-paced breathing (PB). The power spectral density (PSD), phase-locking value (PLV), and brain network characteristics were analyzed for these different conditions.

**Results:**

The results all showed significant differences. The PSD analysis revealed increased low-frequency (*δ* and *θ*) activity during SB and higher high-frequency (*α* and *β*) activity during BH conditions. The PLV analysis demonstrated significant differences in FC between conditions, indicating specific modulation of brain networks by respiratory state. The brain network properties analysis uncovered frequency-specific changes in clustering coefficient (CC), global efficiency (GE), local efficiency (LE), and degree centrality (DC), reflecting alterations in brain network organization. The three-class classifier showed superior performance in the *α* band, suggesting its potential as a biomarker for distinguishing respiratory conditions. Correlation analysis with forced vital capacity (FVC) revealed significant associations between brain connectivity and FVC metrics.

**Discussion:**

These findings highlight the complex interplay between respiratory conditions and brain FC. These findings suggest that controlled and uncontrolled breathing patterns can influence brain network organization, a mechanistic observation that may inform future respiratory-based interventions aimed at enhancing cognitive function, although behavioural or affective outcomes were not assessed here.

## 1 Introduction

The relationship between respiratory conditions and brain functional connectivity (FC) has garnered significant research interest (Yorita et al., 2024; Guo et al., 2025). Emerging evidence indicates that different breathing patterns can modulate brain activity and FC (Anusha et al., 2025). For instance, compared with self-paced breathing (SB), computer-paced breathing (PB) typically using sinusoidal response has been shown to produce changes in blood oxygen level-dependent (BOLD) signals, indicating changes in cerebral blood flow dynamics (Chen et al., 2021). Moreover, the overall delay time in the BOLD response is also significantly different between various respiration rates, suggesting that the rate and pattern of breathing can modulate the efficiency of cerebral vascular responses (Chen et al., 2021). Research has shown that breathing exerts extensive influences on the brain and the body through a complex neural control system, also explored the connections between breathing rhythms and patterns and emotions and cognition (Ashhad et al., 2022). This suggests that respiratory interventions might influence cognitive and emotional processing by modulating brain connectivity. In patients with obstructive sleep apnoea the spatial architecture of EEG synchronization is systematically altered. A marked reduction of symmetrical interhemispheric links that scales with the apnoea hypopnoea index is reported, while intrahemispheric connectivity increases and a left parietal high degree hub emerges (Zhuravlev et al., 2024). Each apnoeic episode triggers transient interhemispheric dominance shifts indexed by delta coherence, phase lag and nonlinear L index, reminiscent of unihemispheric sleep in marine mammals (Rial et al., 2013). Despite this pronounced rewiring, graph theoretical centrality metrics remain surprisingly stable across the night, suggesting that chronic pathology may engage compensatory mechanisms that conserve global network hubs (Sergeev et al., 2025). In contrast, breath-holding (BH) or PB in healthy volunteers offers a model of acute controlled respiratory perturbation that reveals dynamic condition specific shifts in degree centrality (DC), a putative signature of adaptive rather than maladaptive plasticity. The PB further entrains infra slow oscillations of brain potentials to the respiratory cycle, indicating that respiration can act as an endogenous pacemaker for large scale cortical dynamics (Karavaev et al., 2018). Thus, respiration functions as an intrinsic pacemaker for large-scale cortical dynamics.

Research has shown that controlled respiratory cycles could significantly induce alterations in FC in the resting-state network (Park et al., 2022), suggesting that they can differently affect resting brain function (Zelano et al., 2016; Tort et al., 2018; Salimi et al., 2022; Valaei et al., 2024). Research has shown that rhythmic nasal breathing can regulate the oscillations and connectivity of the cerebral cortex (Frohlich et al., 2025). The electroencephalogram (EEG) results have revealed significant differences in heart rate, heart rate variability, and cognitive performance under breathing conditions (Ogoh et al., 2024). This change in connectivity may be linked to enhanced oxygen transport facilitated by nasal breathing, which produces more nitric oxide, a molecule crucial for improving oxygen delivery throughout the body (Mirchandani et al., 2025). Thus, respiratory interventions are presented as a non-pharmacological lever for tuning the brain’s functional architecture.

Recent studies have also highlighted the role of oxygen levels in brain connectivity using fMRI or EEG (Chen et al., 2021; Coronel-Oliveros et al., 2024). Beyond oxygen supply, nasal airflow itself entrains cortical oscillations and FC. Enhanced gamma power and default-mode network connectivity are observed during nasal breathing but not during oral breathing or passive nasal air-puff without active inhalation (Salimi et al., 2023). Spontaneous nasal inhalations phase-lock task-related activity and improve accuracy in non-olfactory cognitive tasks, revealing an evolutionarily preserved mechanism by which inhalation tunes cortical excitability (Perl et al., 2019). In asthma, chronic airway obstruction produces sustained enhancement of both default-mode and salience-network activity, and the magnitude of this enhancement correlates with depression-anxiety scores and reduced pulmonary function (Gholami-Mahtaj et al., 2022). Research has shown that hypoxia (i.e., a situation with low oxygen content) can cause significant changes in the power of the *δ*, *θ*, and *β* frequency bands of the EEG. In an environment with high-altitude hypoxia, over time and after re-oxygenation at lower altitudes, the EEG presents different patterns, indicating that hypoxia reduces brain waves in the lower frequency bands, while hypoxia/re-oxygenation increases brain waves in the higher frequency bands (Zhao et al., 2016). Conversely, adequate oxygen supply is essential for maintaining optimal brain function and cnnectivity. In high-altitude environments, where oxygen levels are naturally lower, individuals often experience cognitive impairments and changes in brain activity, further underscoring the importance of oxygen in brain function (Ramirez-delaCruz et al., 2024). Collectively, these findings indicate that respiration modulates brain networks independent of hypoxia, via rhythmic sensory entrainment and autonomic coupling, providing a direct mechanistic rationale for the frequency-specific connectivity changes observed in the present experimental manipulations.

Beyond healthy physiology, disrupted brain-respiration coupling has been documented in systemic disease models. Many diseases, including cardiovascular, respiratory, and neurological disorders, are related to cerebral hypoxia, which occurs when the brain does not receive adequate oxygen to support normal neuronal function and metabolism. In studies on allergic asthma, it has been shown that inflammatory airway obstruction impairs the control of respiratory rhythm by the limbic system (Dehdar et al., 2022). Some studies have shown that restoring respiratory oscillations can partially reverse the fragmentation of the network in severe hypoxic-ischemic injury (Salimi et al., 2022). In chronic obstructive pulmonary disease (COPD), a recent study utilized graph theory analysis of EEG data to reveal significant differences in various graph indicators of the patients’ brains (Choi and Kim, 2022). This result may be related to chronic hypoxia exposure, which is associated with the pathophysiological mechanism of cognitive deficits in COPD patients. Research has shown that there are statistically significant differences in the congestive heart failure (CHF) statistical topographic maps in the total spectrum and *θ* band (Cacciotti et al., 2023). The EEG abnormalities observed in patients with heart failure bear a strong resemblance to those seen in patients with cognitive impairments. This suggests that there are similarities between the effects of chronic cerebral hypoperfusion caused by neurodegenerative diseases and cardiac conditions on the brain. Research on Alzheimer’s disease indicates the existence of a therapeutic neural regulation pathway that synchronizes with breathing (Salimi et al., 2024). Furthermore, neurodegenerative diseases such as Alzheimer’s and Parkinson’s have also been associated with localized or widespread cerebral hypoxia (Burtscher et al., 2021). Collectively, these studies indicate that respiration-entrained oscillations constitute a global, yet disease-vulnerable, mechanism for maintaining cortical network integrity, motivating the use of voluntary respiratory maneuvers as a probe of network adaptability in health and disease.

Although respiration is known to entrain cortical rhythms, the frequency-specific topology of large-scale brain networks under SB, BH and PB conditions remains poorly defined. In this study, we used high-density EEG to compare FC and graph metrics across SB, BH and PB, hypothesising that these distinct respiratory manoeuvres would differentially modulate *δ-β* band coherence and that the strength of association between FC and FVC would vary across conditions, providing a mechanistic link between breathing physiology and cortical communication.

## 2 Materials and methods

### 2.1 Subjects

EEG data were acquired with the ANT Neuro’s eegoTMmylab system comprising an eego amplifier and a WaveguardTM electrode cap. A transparent, water-soluble conductive gel was applied to each electrode to maintain scalp-electrode impedance below 20 kΩ. Data were digitised continuously at a sampling rate of 500 Hz with the eego amplifier (32-channel configuration) and stored for subsequent offline analysis. A total of 47 right-handed health subjects (19 males, 28 females; age range of 18–28 years, interquartile range 4.5 years) with no self-reported acute or chronic respiratory disorders were enrolled after providing written informed consent for unrestricted publication of the collected data. Anthropometric measures including height, weights, and FVC were available for 35 subjects (13 males, 22 females); the remaining 12 individuals did not undergo these assessments owing to logistical constraints.

### 2.2 Breath-holding tasks

**FIGURE 1:**
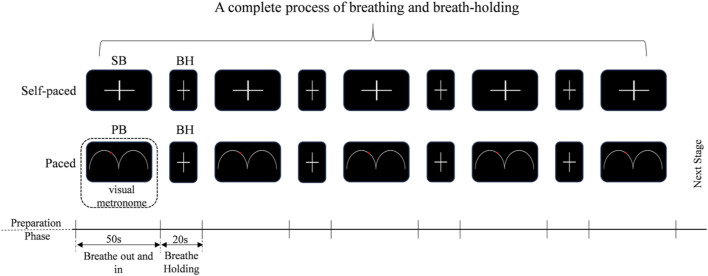
BH tasks design. SB: self-paced breathing, BH: breath-holding, the subjects are required to hold their breath until the word “Hold breath” disappears, and then they should breathe according to their own breathing patterns. PB: computer-paced breathing, the subjects are required to hold their breath until the word “Hold breath” disappeared, and then they adjusted their breathing according to the balls on the screen.

This study used the end-inspiration BH tasks. In addition to SB, PB rates (12 breaths per minute, 0.20 Hz) were also employed. The experimental paradigm was described in Figure 1. The subjects were instructed to control the rhythm of breathing according to the video metronome, that is, the movement track of the ball on the screen, ‘Breathe out’ when the ball was on the uphill track and ‘Breathe in’ on the downhill track. For SB, subjects were instructed to breathe at their own pace. Normally, adults breathe 12–20 times a minute (SB, 0.20Hz - 0.33 Hz) at rest. The self-paced run was always performed at first to avoid the influence of PB (Chen et al., 2021). Then, a text ‘Hold your breath’ was shown on the screen that the subject to hold their breath, using end-inspiration BH. They were required to hold their breath until the word ‘Hold breath’ disappeared and then they adjusted their breathing according to the balls on the screen. Subjects were familiarized with both the computer-paced and self-paced tasks prior to entering the EEG scanner. Each block consisted of 50 s of computer-paced (or self-paced) breathing, alternating with 20 s of BH. These blocks were repeated four times per run, and ended with another 50 s of breathing block, for a total run time of 330 s. Therefore, three experimental blocks were delineated: SB, BH and PB (visual metronome). Throughout all runs, subjects were instructed to breathe at a comfortable depth. In this study, due to the absence of capnography or pulse-oximetry equipment, end-tidal CO_2_ (EtCO_2_) and peripheral oxygen saturation (SpO_2_) were not recorded during or after BH epochs. Respiratory activity was monitored solely by the experimenter’s visual observation; no pneumography, nasal thermistor, belt or capnography was recorded, and therefore exact respiratory rate or breath-by-breath compliance cannot be reported.

### 2.3 EEG data preprocessing

**FIGURE 2:**
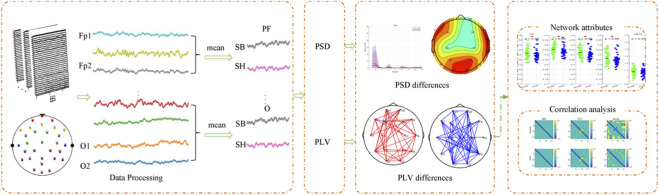
A flow chart of BH-EEG. First, the raw EEG recordings were data processing to obtain the cleaned, average time-series for every channel. Subsequently, PSD differences and PLV connectivity differences were computed between conditions. Finally, based on the functional connection differences of PLV, the differences in network attributes are determined, and correlation anslysis are conducted.

All preprocessing steps were performed using EEGLAB 2025.0.0, an interactive MATLAB toolbox dedicated to the analysis of continuous and event-related electroencephalographic as well as other electrophysiological recordings (Brunner et al., 2013). All the EEG data were preprocessed using standard analysis consisting of the following steps (Yu et al., 2024): (1) Eye movement artifacts removal by the ICA method (Liu et al., 2024); no interpolation was applied and no entire trials or subjects were rejected. (2) 1–45 Hz (including *μ* rhythms and *β* rhythm) bandpass filtering on the EEG time series data. (3) Artifact trail elimination: channels exceeding ±100 µV in >20% of epochs were discarded, leaving a fixed 16-channel montage (Fp1, FP2, F3, F4, F7, F8, T7, T8, C3, C4, P3, P4, P7, P8, O1, O2) (Kang, 2018; Liu et al., 2024); no spatial interpolation was used. For detailed information, refer to Supplementary Table S1. (4) [-200 m, 0 m] baseline correlation was done to compare the effects of stimulating events on brain activity. (5) Frequency division: *δ* (1–4 Hz), *θ* (4–8 Hz), *α* (8–13 Hz), *β* (13–30 Hz); the *γ* band (31–45 Hz) was excluded owing to residual electromyogenic and ocular artifacts that ICA could not fully suppress. Finally, All 47 subjects retained the complete 330 s recording after preprocessing, ensuring equal epoch numbers across SB, BH and PB conditions. The workflow of BH-EEG is shown in Figure 2, including data preprocessing, power spectral density (PSD), phase-locking value (PLV), brain network attributes, and correlation analysis of FVC. The specifics of these steps are described in the following sections.

### 2.4 Power spectral density (PSD)

The power spectral density (PSD) of each preprocessed data block was estimated using Welch’s modified periodogram method. Each block was windowed with a 128-sample Hamming window, overlapped by 50%, and zero-padded to 256 points; the discrete Fourier transform was then averaged across overlapping segments to yield a spectral resolution of 1 Hz (Walters et al., 2025). Log-transformed power spectrum was extracted across four canonical bands. Channel-wise mean PSD values were obtained for each block (SB, BH and PB) and subsequently averaged across epochs within each participant.

### 2.5 Phase-locking value (PLV)

To calculate the FC among all available pairs of electroencephalogram electrodes, the PLV method was adopted (Jahromy et al., 2019). The PLV was computed for each preprocessed data block using the Hilbert transform. For every channel pair, instantaneous phase time-series were extracted from the analytic signal, and phase differences were calculated sample-by-sample (Gonuguntla and Kim, 2020). The absolute value of the mean resultant vector across time yielded the PLV for each pair; values range from 0 (no phase coupling) to 1 (perfect locking). PLV was averaged within four canonical frequency bands and across epochs for each block (SB, BH, PB), producing participant-specific, channel-pair connectivity matrices.

### 2.6 Graph theory

Weighted adjacency matrices were used without binarisation or density thresholding; all PLV values were retained to preserve continuous connectivity information. The clustering coefficient (CC) was calculated for every node using the geometric mean of edge weights within each triplet and averaged across the network to yield the global CC. The local efficiency (LE) was defined analogously for the subgraph induced by each node’s immediate neighbors and then averaged across nodes. The global efficiency (GE) was obtained as the inverse of the average shortest weighted path length between all node pairs. The DC was computed as the mean of the weighted degree across all electrodes. Future studies will employ surrogate-based null models across multiple density levels (10%–40%) to verify the robustness of CC, LE, GE and DC findings.

### 2.7 Correlation analysis

For each frequency band, the 16 × 16 PLV matrices obtained during both the breathing and breath-hold phases were reshaped into 256-dimensional vectors (upper-triangular elements). Pearson correlations were computed between each of the 256 PLV values and the FVC data for the 35 subjects. To control for multiple comparisons, *p*-values were corrected across the 256 × 3 = 768 tests using the Benjamini–Hochberg false-discovery-rate (FDR) procedure at *α* = 0.01.

### 2.8 Feature selection and classification

The symmetric 16 × 16 connectivity matrices were vectorized by extracting the upper-triangular elements (excluding the diagonal), yielding 120 unique functional connections per participant and frequency band. To identify the minimal set of functional connections that optimally discriminates the three respiratory blocks (SB, BH, PB), we first concatenated the 47 × 120 connectivity matrices across the four canonical frequency bands. Within each band, ReliefF was applied to rank all 120 features according to their relevance to the three-class label, and the top 30 connections were retained. These 30 selected features served as input to a lightweight fully-connected neural network implemented in MATLAB. The architecture comprised a feature-input layer (dimensionality = 30), two successive dense layers (64 and 32 neurons) with Rectified Linear Unit activation, each followed by a 30% dropout layer, and a final softmax layer yielding three class probabilities. The model was trained for 200 epochs using the Adam optimizer (initial learning rate 0.001, L2-regularization 0.001, mini-batch size 16). Training and validation were performed on the same 47-subject dataset; classification accuracy and Cohen’s κ were computed from the network predictions. The 47-subject dataset was randomly divided into ten stratified folds preserving class proportions; nine folds were used for training and the remaining one for testing. This procedure was repeated ten times and the reported accuracy and κ value represent the mean across these ten iterations.

## 3 Results

### 3.1 The differences in PSD between controlled and uncontrolled breathing

**FIGURE 3:**
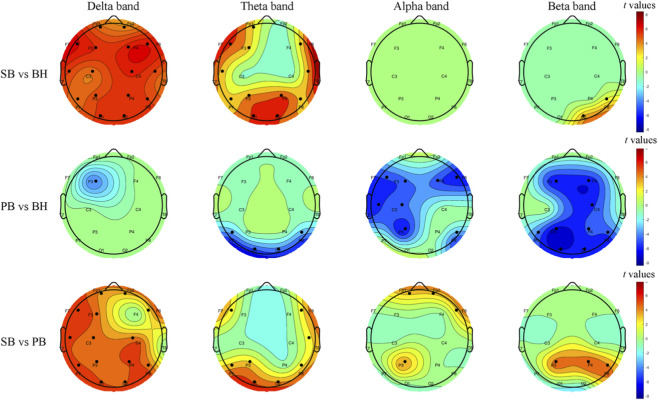
Topographic *t*-maps illustrate group-level PSD differences between respiratory conditions, corrected for multiple comparisons with family-wise error (FWE, *p* < 0.01). Each row corresponds to a paired contrast: SB vs. BH, PB vs. BH and SB vs. PB. Columns represent frequency bands (*δ*, *θ*, *α*, *β*). Warm colors denote higher PSD in the first block; cool colors denote higher PSD in the second block. Black dots indicate electrodes with significant differences. The scalp layout follows the 1–16 system.

We performed two-sample t-tests (FWE *p* < 0.01) to explore the differences between each pair of blocks, and the results are shown in Figure 3. In the comparison between SB and BH, significant differences were found in the low-frequency bands, including the *δ* and *θ* bands. Specifically, SB exhibited higher PSD than BH in these bands, particularly in the frontocentral region (F3, F4) and the central parietal area (C3, C4). In contrast, minimal differences were observed in the high-frequency bands, such as the *α* and *β* bands. Notably, in the *α* band, no significant differences were found, while in the *β* band, SB showed higher PSD than BH at channels P8 and O2. When comparing PB and BH, there was no statistical differences in the low-frequency bands. However, in the high-frequency bands, PB showed significantly lower PSD than BH. For instance, in the *θ* band, PB exhibited lower PSD than BH at channels P7, P8, O1, and O2. In the *α* band, PB demonstrated more pronounced suppression in the frontal lobe (F3, F4, F7, F8) and the left hemisphere. In the *β* band, PB showed more significant suppression across the entire brain. In the comparison between SB and PB, more significant differences were found in the low-frequency bands. In the *δ* band, except for channels F4 and T8, SB showed higher PSD than PB. The regions of significant differences in the *θ* and *α* bands were relatively small, but in the *β* band, SB exhibited higher PSD than PB in the parietal lobe (P3, P4, P8). For detailed information, refer to Supplementary Table S2.

### 3.2 The differences in FC between controlled and uncontrolled breathing

**FIGURE 4:**
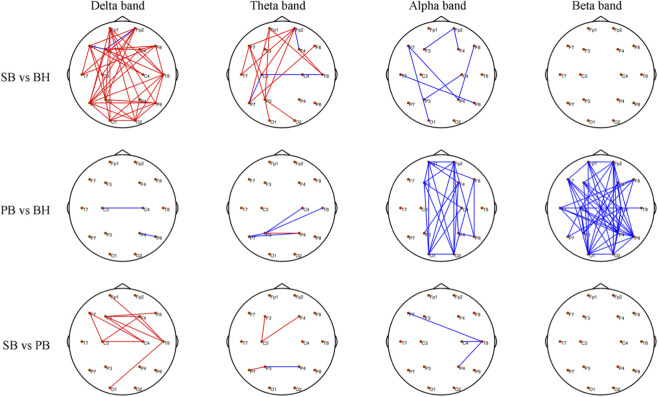
Group-level PLV differences across three contrasts (SB vs. BH, PB vs. BH, SB vs. PB) are depicted via circular topographic t-maps. Each row corresponds to one contrast, and each column to a frequency band (*δ*, *θ*, *α*, *β*). Red lines indicate significantly higher PLV in the first-listed block; blue lines indicate higher PLV in the second-listed block. Nodes represent EEG electrode positions (1–16 system). Results are FWE-corrected (*p* < 0.01).

We analyzed EEG functional networks under different breathing conditions using PLV-based inter-regional connectivity matrices. Figure 4 shows two-sample t-test results (FWE-corrected, *p* < 0.01) for PLV differences across three blocks pairs (SB and BH, PB and BH, SB and PB) and four frequency bands (*δ*, *θ*, *α*, *β*). Each row represents a block pair, and each column represents a frequency band. The figure uses colors and lines to indicate significant connectivity differences: red lines mean higher PLV in the first block (e.g., SB), and blue lines mean higher PLV in the second block (e.g., BH). When comparing SB and BH, significant differences were found in the low-frequency bands (*δ* and *θ*). In these bands, SB exhibited stronger connectivity in the frontal (Fp1, Fp2) and prefrontal (F7, F3, F4, F8) regions, with the most notable enhancements in the *δ* band. This suggests a stronger low-frequency functional connection during SB. In the *α* band, SB showed a slight reduction in connectivity. No significant differences were observed in the *β* band, indicating similar high-frequency connectivity patterns between the two blocks. In the comparison of PB and BH, no significant differences in FC were found in the low-frequency bands (*δ* and *θ*). However, in the high-frequency bands (*α* and *β*), BH demonstrated significantly stronger connectivity than PB. This was particularly evident in regions such as Fp1, Fp2, F3, F4, C3, C4, P3, P4, O3, and O4, with the most prominent enhancements observed in the *β* band. When comparing SB and PB, the most significant differences were found in the *δ* band, where SB exhibited stronger connectivity in regions such as F3, F4, C3, C4, and T8. In the *θ* and *α* bands, SB showed fewer and weaker connections compared to PB. No significant differences were found in the *β* band. For detailed information, refer to Supplementary Table S3.

### 3.3 Brain network properties

**FIGURE 5:**
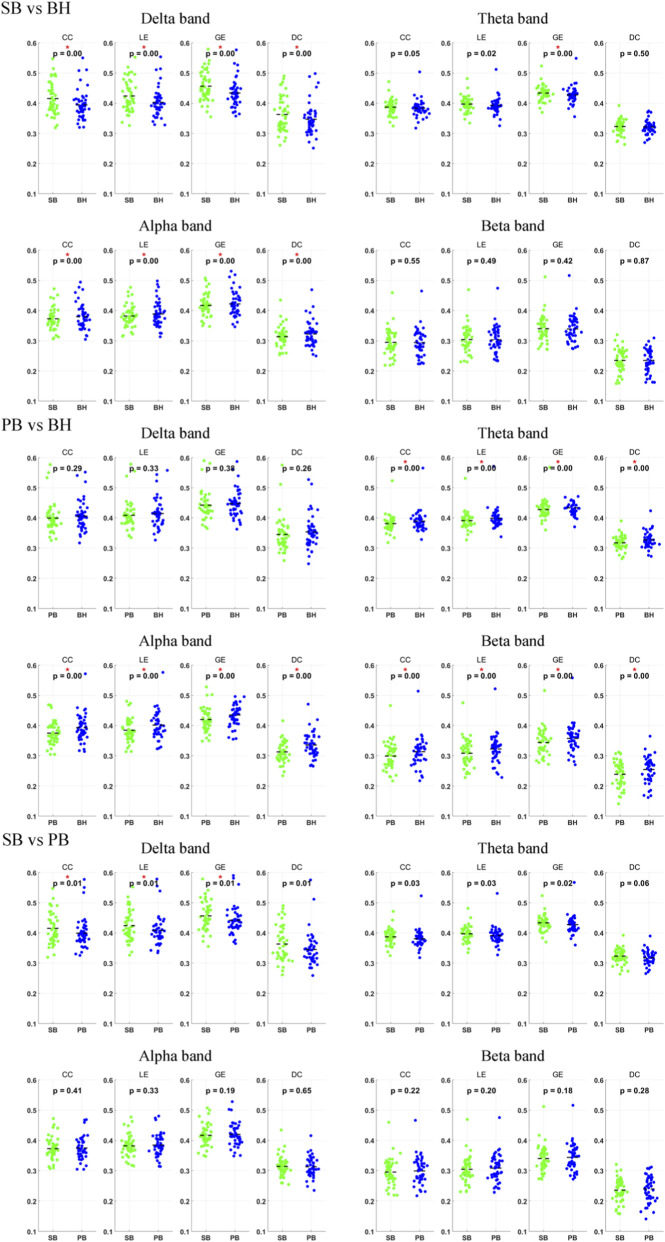
Group-level network properties across three contrasts (SB vs. BH, PB vs. BH, SB vs. PB) are depicted for four metrics: CC, LE, GE and DC. Green indicates the first block (e.g., SB), and blue indicates the second block (e.g., BH). Red asterisks highlight significant differences (t-test, *p* < 0.01).

We examined the frequency-specific modulation of network properties across different breathing conditions. We calculated the two-sample t-test results (*p* < 0.01) for four network properties (CC, LE, GE, DC) across three condition pairs (SB and BH, PB and BH, SB and PB) and four frequency bands (*δ*, *θ*, *α*, *β*). Figure 5 presents these results, green indicates the first block (e.g., SB), while blue indicates the second block (e.g., BH), and red asterisks highlight significant differences (t-test, *p* < 0.01). For the comparison between SB and BH, significant differences in network properties were observed in the *δ* and *α* bands. In the *θ* band, only GE showed significant differences, and no significant differences were found in the *β* band. In the comparison between PB and BH, no significant differences were found in the *δ* band. However, significant differences in all network properties were observed in the *θ*, *α* and *β* bands. For the comparison between SB and PB, significant differences were found only in the *δ* band, specifically in CC, LE and GE. No significant differences were found in the other frequency bands.

### 3.4 Correlation between FCs and FVC in the PB across frequencies

We systematically analyzed the correlation between FC (PLV) across different breathing conditions and FVC, with the results presented in Table 1. The correlation analysis between FC and FVC showed that under different breathing conditions, there were significant correlations between FVC and the connection strength of specific brain regions across multiple frequency bands. In the *δ* band, channels like C4, T8, F3, and P7 were frequently involved. In the *θ* band, the correlation was significant for channels such as C4, T8, P7, and O1. In the *α* band, the connection of channels T8, O1, P7, and O2 stood out. In the *β* band, the correlation was more evident for channels T7, O1, P3, and O2. Notably, channels T8, O1, P7, and C4 repeatedly appeared across multiple bands, indicating they may be reliable indicators of FVC-related brain FC. Further analysis revealed that the frequency of these channels and their correlation patterns varied across different breathing conditions (SB, BH, PB), suggesting that breathing states modulate the relationship between FVC and brain FC. These findings offer a fresh perspective on the complex relationship between FVC and brain FC, showing that certain channels maintain consistent correlation patterns across frequency bands and breathing conditions, with correlation coefficients ranging from 0.43 to 0.53, indicating moderate correlation strength.

**TABLE 1:** The correlation coefficient between FC and FVC (FDR *p* < 0.01).

Band	Respiratory condition	FC	*r*	*p*-value
Delta band	SB	C4-T8	0.44	0.0080
	BH	F3-T8	0.43	0.0097
	PB	F3-T8	0.53	0.0011
		F3-P7	0.53	0.0010
Theta band	SB	C4-T8	0.51	0.0016
		C4-P7	0.47	0.0042
	BH	C4-T8	0.48	0.0034
		C4-P7	0.49	0.0028
		T8-P8	0.45	0.0065
	PB	F7-P4	0.45	0.0073
		T8-O1	0.45	0.0074
Alpha band	SH	F8-O1	0.44	0.0089
		T8-O1	0.45	0.0072
		P7-O2	0.45	0.0062
Beta band	SB	T7-O1	0.49	0.0027
		P3-O1	0.43	0.0099
		T8-O2	0.44	0.0081
		P7-O2	0.47	0.0041
	BH	Fp1-P3	0.44	0.0084
		T7-O1	0.43	0.0094
		P3-O1	0.45	0.0062
		T8-O2	0.44	0.0078
		P7-O2	0.48	0.0033
	PB	T7-O1	0.53	0.0010
		P3-O1	0.44	0.0075

### 3.5 Classification

Table 2 shows the results obtained using a three-class classifier to extract features and classify different conditions. The table illustrates the classification outcomes for various frequency bands. The classifier performed optimally in the *α* band, achieving an accuracy of 85.11% and a κ value of 0.77, indicating high classification consistency. In the *δ* band and the *β* band, the accuracy rate reached 78.72% in both cases, and the κ value was 0.68 in each case. In the *θ* band, the accuracy was 74.47% and the κ value was 0.61. Overall, the classifier demonstrated superior performance in the *α* band compared.

**TABLE 2:** The classification results of the 3-category using SVM classifier in different frequency bands.

Band	Frequency	Accuracy	κ
Delta	1–4 Hz	78.72%	0.68
Theta	4–8 Hz	74.47%	0.61
Alpha	8–13 Hz	85.11%	0.77
Beta	13–30 Hz	78.72%	0.68

## 4 Discussion

In this study, we investigated the impact of different respiratory conditions on brain FC using EEG recordings. The PSD analysis revealed distinct patterns across frequency bands and conditions, highlighting differences in brain activity during various breathing states. The PLV analysis demonstrated significant differences in FC between conditions, indicating condition-specific modulation of brain networks. Brain network properties analysis uncovered frequency-specific changes in CC, GE, LE, and DC, reflecting alterations in brain network organization. The three-class classifier showed superior performance in the *α* band, suggesting it as a potential biomarker for distinguishing respiratory conditions. Finally, the correlation analysis between FC and FVC revealed a significant association with the brain connectivity indicators, highlighting the relationship between lung volume and brain function.

Our study reveals distinct PSD patterns across respiratory states, providing a pivotal tool for elucidating brain-activity dynamics under different breathing conditions (Zhang et al., 2023). During SB, higher PSD in the *δ* band was observed compared to BH, which may be associated with the brain’s regulatory activities during normal breathing. In contrast, BH exhibited elevated PSD in higher-frequency bands (*θ*, *α*, and *β*) relative to SB, suggesting a subtle shift in brain activity towards higher frequencies during BH (Sinha et al., 2020). BH showed significantly higher PSD across all frequency bands compared to PB, with a notable increase in the *α* band at the Fp2 channel, indicating that BH may enhance overall brain activation (Mullins et al., 2021). This study found that the PSD level of SB in the low-frequency range was higher than that of PB, while the PSD level of PB in the high-frequency range was higher than that of SB. These findings collectively highlight the complex relationship between respiratory conditions and brain FC, as evidenced by PSD variations across different breathing states (Girin et al., 2021). These results are consistent with previous studies indicating that PSD changes reflect functional brain activity under varying physiological states, particularly in cognitive and emotional contexts (Prasad et al., 2023; Zhang W. et al., 2024). These studies collectively support the notion that PSD analysis is a valuable tool for understanding the neurophysiological mechanisms underlying respiratory regulation of brain networks.

Furthermore, our pronounced PLV patterns across breathing states serve as a key instrument for clarifying large-scale connectivity dynamics during respiratory variations (Wang et al., 2023). During SB, stronger low-frequency connections (*δ* and *θ* bands) were observed in the frontal (Fp1, Fp2) and prefrontal (F7, F3, F4, F8) regions compared to BH, which may be associated with the brain’s regulatory activities during normal breathing. The *δ* band along the frontal midline is closely related to cognition and executive control during psychological tasks, such as memory processing, arithmetic problem-solving, or sustained attention (Chander et al., 2016). And the increase in PLV occurred in the left parieto-occipital-temporal region and the medial frontal region at the *δ* band, as well as in the *α* band in the medial frontal region (Han et al., 2022). In contrast, BH demonstrated significantly stronger high-frequency connections (*α* and *β* bands) than PB, particularly in regions such as Fp1, Fp2, F3, F4, C3, C4, P3, P4, O3, and O4. This suggests that BH may enhance overall brain activation and connectivity (Chen et al., 2021). SB was found to have stronger low-frequency connections than PB, while PB had stronger high-frequency connections than SB. BH demonstrated stronger high-frequency connections compared to BH. These findings collectively highlight the complex relationship between respiratory conditions and brain FC, as evidenced by PLV variations across different breathing states (Migliorelli et al., 2019; Zhang C. et al., 2024). These results are consistent with previous studies indicating that PLV changes reflect functional brain connectivity under varying physiological states, particularly in cognitive and emotional contexts (Wu et al., 2023; Hu et al., 2024). These studies collectively support the notion that PLV analysis is a valuable tool for understanding the neurophysiological mechanisms underlying respiratory regulation of brain networks.

Our analysis of brain network properties revealed significant modulations in CC, LE, GE, and DC in different breathing conditions. These condition-specific shifts reflect re-configuration between local segregation (higher CC, LE) and global integration (higher GE, DC) rather than intrinsic improvement, their functional valence awaits behavioural validation. In our study, comparing SB vs. BH and PB vs. BH revealed significant network property differences. For SB vs. BH, significant variations emerged in the *δ* and *α* bands, with only GE showing differences in the *θ* band and no differences in the *β* band. In contrast, for PB vs. BH, while no differences were found in the *δ* band, the *θ* band’s σ showed no significant differences, and all four network properties significantly differed in the *α* and *β* bands. These findings indicate that network properties in the *θ* and *β* bands are particularly sensitive to changes in respiratory conditions. The *θ* band is closely tied to cognitive processes such as memory and attention (Tan et al., 2024), while the *β* band is associated with motor and cognitive functions, reflecting active information processing and sustained attention (Leunissen et al., 2022). Some studies have emphasized the importance of the *θ* and *β* bands in the integration of complex sensory and motor processes (Bottcher et al., 2023), which is consistent with our research results. In addition, comparing SB vs. PB revealed significant differences in network properties only in the *δ* band, specifically in CC, LE, and GE, with no significant differences in other frequency bands. The *δ* band is closely associated with deep sleep, meditation, and states of relaxation. It reflects the brain’s ability to maintain basic physiological functions and is often linked to emotional processing and stress regulation (Allegretta et al., 2024). Research has shown that OSA patients preserve centrality despite extensive connectivity rewiring, whereas healthy volunteers exhibit significant frequency specific modulation of DC during short BH or PB epochs (Sergeev et al., 2025). This discrepancy points to a fundamental difference between chronic compensatory stability and acute task evoked flexibility. Future studies should also examine whether loss of infra slow phase locking between respiration and EEG, previously demonstrated during PB (Karavaev et al., 2018), marks an early transition from adaptive to pathological network states in respiratory disease. Consistent with a recent mini-review on maximal apnoea protocols (Ribeiro et al., 2024), the present BH epochs produced an *α* band reduction that was detectable in both untrained volunteers and would likely be accentuated in apnea-trained free divers, this supports the view that voluntary cessation of breathing provides a reproducible model for studying cortical responses to respiratory distress. Research has shown that the topological structure of the brain networks in patients with COPD changes. In the *α* band, the CC, LE, GE, and DC of COPD patients are all lower than those of the control group, LE show differences mainly in the occipital parietal region (Choi and Kim, 2022). These bands are crucial for understanding how respiratory conditions modulate brain network properties. By examining these network properties, we gain insights into the brain’s ability to adapt and respond to different respiratory states, which is essential for understanding the neurophysiological mechanisms underlying respiratory regulation of brain networks.

Our study uncovered significant links between FC and FVC across various breathing states and frequency bands. As a fundamental spirometric index, FVC reflects the maximum volume of air that can be forcibly exhaled after full inspiration. In the present study, several cortical channels exhibited robust, frequency-specific correlations with FVC across SB, BH and PB conditions. These reproducible associations indicate that inter-individual differences in lung volume are linked to systematic variations in EEG FC. The correlation analysis between FC and FVC showed significant associations across different breathing conditions and frequency bands. Robust, moderate correlations (*r* = 0.43–0.53, FDR *p* < 0.01) were observed between *α/β* connectivity and FVC in 35 participants; effect sizes exceed Cohen’s κ threshold for a medium effect. FVC here indexes maximal mechanical lung capacity rather than cardiorespiratory fitness *per se*, suggesting that larger vital capacity may facilitate efficient diaphragmatic oscillations that entrain cortical networks. These correlations were stable across SB, BH and PB conditions, but the smaller FVC subset (n = 35 vs. 47) limits power; future studies with full pulmonary-function panels and fitness metrics are needed to confirm whether the relationship extends to aerobic capacity or is specific to static lung volumes. Our research also found significant correlations between FVC and brain FC in regions such as C4, T7, O1, and P3. The study on COPD patients revealed altered RSFC in brain regions that overlap with key areas from our research (Han et al., 2025). Specifically, resting-state FC was reduced in the right superior occipital gyrus (O2) and increased in the left superior temporal sulcus (T7) and right precentral gyrus (C4). These shared regions are crucial for respiratory control and sensory processing. Both studies highlight the relationship between respiratory function/conditions and brain connectivity, suggesting that FVC is closely associated with brain network properties in these areas. Research indicates that higher cardiovascular health is linked to enhanced connectivity in the default mode and salience networks, and better sleep is associated with increased connectivity in multiple networks, including the default mode, executive control, and salience networks (Wing et al., 2024). Thus, a controlled breathing pattern may have a negative impact on cardiovascular and respiratory functions as well as sleep quality by reducing the functional connections within these networks. Although we observed only acute EEG effects, the reduced connectivity within the default mode and salience networks during PB parallels the diminished connectivity reported in individuals with lower cardiorespiratory fitness and poor sleep quality. This suggests that repeated or prolonged periods of controlled breathing could, by persistently down-regulating these network connections, contribute to similar negative cardiovascular and sleep outcomes, providing a mechanistic hypothesis for future longitudinal studies.

Our three-class classifier analysis shows that the *α* band is optimal for distinguishing different respiratory conditions, with the highest accuracy and consistency. The *α* band of EEG is closely related to the relaxed state of the brain, especially when the eyes are closed, and the alpha activity in the visual cortex is enhanced, reflecting the inhibitory neuronal activity (London et al., 2022). This may be related to our BH tasks design. When attention needs to be focused, the power of alpha waves decreases, indicating that the brain shifts from a relaxed state to a state of focus (Pascucci et al., 2025). In addition, *α* oscillations are associated with the default mode network (DMN) (Pusil et al., 2023), which is active in the resting state and involved in mental processes such as self-reflection and cognitive introgression. Although the present design did not include psychometric or affective measures, the *α*-band network modulation observed here might offer a putative marker for future studies examining whether paced-breathing protocols can benefit emotional regulation.

This research holds significant implications for understanding the neurophysiological mechanisms underlying respiratory regulation of brain networks. Although, in the present design did not include a continuously free-breathing control block with identical visual stimulation, and the absence of simultaneous respiratory recordings prevents us from disentangling the specific contribution of respiratory modulation from that of attention shifts or visual-input changes. However, by elucidating how controlled and uncontrolled breathing patterns affect brain connectivity, in the future we may be able to develop targeted respiratory intervention measures to enhance cognitive functions and emotional regulation abilities. Without SpO_2_ or, EtCO_2_ data we cannot quantify the degree of hypoxia or hypercapnia produced by our 20-s breath-hold epochs; consequently, any discussion linking observed EEG changes to hypoxic stress remains speculative. Future studies should include simultaneous CO_2_ and O_2_ monitoring to disentangle the contribution of chemoreceptor-driven signals from the mechanical/phase-locked effects of respiration itself. Furthermore, this study may provide insights into how oxygen levels and high-altitude environments interact with respiratory patterns to influence brain function. Our findings could contribute to the development of respiratory-based therapies for conditions where brain connectivity is compromised, such as in neurological and psychiatric disorders. Additionally, understanding the relationship between respiratory patterns and brain connectivity may offer new strategies for improving cognitive performance and overall brain health in high-altitude settings or conditions with reduced oxygen availability.

## 5 Conclusion

Our study used EEG recordings to explore how different respiratory conditions modulate brain FC, revealing distinct patterns in PSD, PLV, and brain network properties. We observed higher low-frequency activity during spontaneous breathing and higher high-frequency activity during BH, with PLV analysis showing significant differences in FC across conditions. Brain network properties analysis highlighted frequency-specific changes, particularly in the *α* band, which performed best in classification. Correlations between brain connectivity and lung volume like FVC were also significant. These results provide a mechanistic basis for future trials that test whether respiratory-based interventions improve cognition once behavioural benefits are confirmed.

## Data Availability

The data that support the findings of this study will be made available on request from the corresponding authors. The data are not publicly available due to privacy or ethical restrictions. Requests to access the datasets should be directed to Kai Chen, chenkaixhu@163.com.
