# Supplementary material for: Dynamic brain network modulation by paced breathing and breath-holding: an EEG-based functional connectivity study
Source: Front Physiol. 2025 Dec 4;16:1722715. doi: 10.3389/fphys.2025.1722715 (PMC12711548; doi:10.3389/fphys.2025.1722715)
Supplement: Supplementary file 1 [file Table1.docx]

**Table S1** The channels and the corresponding brain regions.

|  | Channel | Brain region |
| --- | --- | --- |
| 1 | Fp1 | Left Pre-frontal |
| 2 | Fp2 | Right Pre-frontal |
| 3 | F7 | Left Frontal |
| 4 | F3 | Left Frontal-central |
| 5 | F4 | Right Frontal-central |
| 6 | F8 | Right Frontal |
| 7 | T7 | Left Temporal |
| 8 | C3 | Left Central |
| 9 | C4 | Right Central |
| 10 | T8 | Right Temporal |
| 11 | P7 | Left Parietal |
| 12 | P3 | Left Parietal-central |
| 13 | P4 | Right Parietal-central |
| 14 | P8 | Right Parietal |
| 15 | O1 | Left Occipital |
| 16 | O2 | Right Occipital |

**Table S2** The differences (*t*-values, FWE *p* < 0.01) in PSD for the three situations.

|  | SB vs BH | | | | PB vs BH | | | SB vs PB | | | |
| --- | --- | --- | --- | --- | --- | --- | --- | --- | --- | --- | --- |
| Channel | *δ* | *θ* | *β* | *δ* | *θ* | *α* | *β* | *δ* | *θ* | *α* | *β* |
| Fp1 | 4.71 | 3.77 |  |  |  |  |  | 4.65 |  | 3.92 |  |
| Fp2 | 3.98 |  |  |  |  |  |  | 4.59 |  | 4.14 |  |
| F7 | 6.72 | 7.01 |  |  |  | -5.01 |  | 6.25 | 4.45 |  |  |
| F3 | 5.98 | 4.09 |  | -3.75 |  | -4.67 | -5.18 | 5.51 |  |  |  |
| F4 | 6.45 |  |  |  |  | -4.87 | -5.12 |  |  |  |  |
| F8 | 6.09 | 7.52 |  |  |  | -5.83 |  | 5.55 | 5.22 | 4.68 |  |
| T7 | 5.44 | 4.94 |  |  |  | -5.08 |  | 5.65 |  |  |  |
| C3 | 4.52 |  |  |  |  | -3.86 |  | 4.96 |  |  |  |
| C4 | 5.83 |  |  |  |  |  | -4.43 | 5.39 |  |  |  |
| T8 | 4.60 | 7.56 |  |  |  |  |  |  | 4.06 |  |  |
| P7 | 4.93 | 3.87 |  |  | -5.04 |  | -4.36 | 5.71 | 5.37 |  |  |
| P3 | 5.36 | 5.05 |  |  |  | -5.64 | -6.80 | 5.21 | 4.01 | 4.01 | 4.87 |
| P4 | 5.68 | 6.13 |  |  |  |  | -4.54 | 5.41 |  |  | 5.64 |
| P8 | 5.61 | 3.94 | 3.72 |  | -4.47 | -4.35 | -5.37 | 5.62 | 4.50 |  | 4.93 |
| O1 | 5.59 | 6.08 |  |  | -4.69 |  | -5.92 | 5.42 | 5.69 |  |  |
| O2 | 5.69 | 6.00 | 3.83 |  | -3.98 |  | -5.36 | 5.34 | 4.83 |  |  |

**Table S3** The differences (*t*-values, FWE *p* < 0.01) in FC for the three situations.

|  | FC | *t*-value | FC | *t*-value | FC | *t*-value | FC | *t*-value |
| --- | --- | --- | --- | --- | --- | --- | --- | --- |
|  | *δ* | | *θ* | | *α* | | *β* | |
| SB  vs  BH | Fp2-F7 | 8.09 | Fp2-F7 | 6.12 | Fp2-F4 | -5.28 |  |  |
|  | Fp2-F4 | -4.58 | Fp2-F4 | -6.37 | Fp2-F4 | -4.99 |  |  |
|  | F7-F4 | -5.25 | Fp2-T7 | 6.92 | C4-P3 | -5.21 |  |  |
|  | F4-F4 | 8.47 | F7-T7 | 5.01 | F7-P4 | -4.97 |  |  |
|  | F4-F8 | 5.71 | Fp2-C3 | 6.80 | F8-P4 | -4.92 |  |  |
|  | F4-F8 | 4.55 | F7-C3 | 6.73 | T7-P8 | -5.41 |  |  |
|  | Fp2-T7 | 9.13 | Fp1-T8 | 5.82 | F7-O1 | -4.79 |  |  |
|  | F7-T7 | 9.06 | F4-T8 | 5.45 |  |  |  |  |
|  | F8-T7 | 5.85 | C3-T8 | -5.43 |  |  |  |  |
|  | Fp2-C3 | 7.36 | Fp1-P7 | 4.73 |  |  |  |  |
|  | F7-C3 | 7.85 | F8-P7 | 4.94 |  |  |  |  |
|  | F7-C4 | 5.03 | C3-P7 | -7.21 |  |  |  |  |
|  | F4-C4 | 4.65 | Fp1-P3 | 5.32 |  |  |  |  |
|  | Fp1-T8 | 9.00 | Fp2-P3 | 6.39 |  |  |  |  |
|  | F4-T8 | 8.66 | F7-P3 | 5.82 |  |  |  |  |
|  | F4-T8 | 8.88 | P3-O1 | 6.93 |  |  |  |  |
|  | F8-T8 | 7.36 | P3-O2 | 5.71 |  |  |  |  |
|  | Fp1-P7 | 8.06 |  |  |  |  |  |  |
|  | Fp2-P7 | 5.91 |  |  |  |  |  |  |
|  | F4-P7 | 6.11 |  |  |  |  |  |  |
|  | F4-P7 | 5.66 |  |  |  |  |  |  |
|  | F8-P7 | 8.63 |  |  |  |  |  |  |
|  | T8-P7 | 6.41 |  |  |  |  |  |  |
|  | Fp1-P3 | 6.24 |  |  |  |  |  |  |
|  | Fp2-P3 | 7.63 |  |  |  |  |  |  |
|  | F7-P3 | 7.53 |  |  |  |  |  |  |
|  | C3-P3 | 4.60 |  |  |  |  |  |  |
|  | Fp1-P4 | 4.81 |  |  |  |  |  |  |
|  | Fp1-P8 | 7.61 |  |  |  |  |  |  |
|  | T8-P8 | 5.30 |  |  |  |  |  |  |
|  | P7-P8 | 5.65 |  |  |  |  |  |  |
|  | P3-P8 | 5.79 |  |  |  |  |  |  |
|  | Fp1-O1 | 7.47 |  |  |  |  |  |  |
|  | T8-O1 | 7.52 |  |  |  |  |  |  |
|  | P7-O1 | 7.18 |  |  |  |  |  |  |
|  | P3-O1 | 8.06 |  |  |  |  |  |  |
|  | P8-O1 | 6.10 |  |  |  |  |  |  |
|  | Fp1-O2 | 6.62 |  |  |  |  |  |  |
|  | T8-O2 | 6.99 |  |  |  |  |  |  |
|  | P7-O2 | 6.78 |  |  |  |  |  |  |
|  | P3-O2 | 6.06 |  |  |  |  |  |  |
|  | P4-O2 | 5.34 |  |  |  |  |  |  |
|  | P8-O2 | 6.77 |  |  |  |  |  |  |
|  | O1-O2 | 7.76 |  |  |  |  |  |  |
| PB  vs  BH | C3-C4 | -4.91 | C4-P3 | -7.23 | Fp1-Fp2 | -5.67 | Fp1-Fp2 | -6.10 |
|  | P4-P8 | -5.15 | T8-P3 | -5.20 | Fp1-F4 | -5.83 | Fp1-F7 | -5.82 |
|  |  |  | P7-P3 | -6.30 | Fp2-F4 | -5.07 | Fp2-F4 | -5.81 |
|  |  |  | P7-P4 | -5.08 | Fp1-F4 | -5.95 | F8-T7 | -5.18 |
|  |  |  | P3-P4 | 4.82 | Fp1-F8 | -4.76 | Fp2-C3 | -6.12 |
|  |  |  |  |  | F4-C4 | -5.87 | F7-C3 | -4.97 |
|  |  |  |  |  | F4-P3 | -5.78 | F8-C3 | -6.80 |
|  |  |  |  |  | Fp2-P4 | -4.55 | Fp2-C4 | -5.38 |
|  |  |  |  |  | Fp2-P8 | -4.93 | F4-C4 | -4.95 |
|  |  |  |  |  | F8-P8 | -5.14 | F4-C4 | -5.12 |
|  |  |  |  |  | P4-P8 | -6.21 | T7-T8 | -5.13 |
|  |  |  |  |  | Fp1-O1 | -5.60 | C3-P7 | -5.56 |
|  |  |  |  |  | Fp2-O1 | -6.01 | C4-P7 | -5.80 |
|  |  |  |  |  | F4-O1 | -5.90 | F4-P3 | -5.40 |
|  |  |  |  |  | F4-O1 | -5.04 | F8-P3 | -4.63 |
|  |  |  |  |  | C4-O1 | -4.91 | T7-P3 | -6.72 |
|  |  |  |  |  | Fp1-O2 | -7.08 | C3-P3 | -5.40 |
|  |  |  |  |  | Fp2-O2 | -7.04 | Fp1-P4 | -4.77 |
|  |  |  |  |  | F4-O2 | -5.45 | Fp2-P4 | -4.86 |
|  |  |  |  |  | F4-O2 | -5.84 | F7-P4 | -5.96 |
|  |  |  |  |  | F8-O2 | -5.20 | F4-P4 | -5.23 |
|  |  |  |  |  | O1-O2 | -4.95 | F8-P4 | -5.93 |
|  |  |  |  |  |  |  | T7-P4 | -5.94 |
|  |  |  |  |  |  |  | C3-P4 | -5.65 |
|  |  |  |  |  |  |  | C4-P4 | -5.62 |
|  |  |  |  |  |  |  | Fp1-P8 | -6.07 |
|  |  |  |  |  |  |  | Fp2-P8 | -7.12 |
|  |  |  |  |  |  |  | F7-P8 | -5.07 |
|  |  |  |  |  |  |  | F4-P8 | -6.51 |
|  |  |  |  |  |  |  | T7-P8 | -6.33 |
|  |  |  |  |  |  |  | C3-P8 | -5.93 |
|  |  |  |  |  |  |  | C4-P8 | -6.08 |
|  |  |  |  |  |  |  | P4-P8 | -9.89 |
|  |  |  |  |  |  |  | Fp1-O1 | -7.11 |
|  |  |  |  |  |  |  | Fp2-O1 | -6.69 |
|  |  |  |  |  |  |  | F7-O1 | -5.11 |
|  |  |  |  |  |  |  | F4-O1 | -5.58 |
|  |  |  |  |  |  |  | F4-O1 | -5.34 |
|  |  |  |  |  |  |  | C3-O1 | -4.62 |
|  |  |  |  |  |  |  | C4-O1 | -6.02 |
|  |  |  |  |  |  |  | P4-O1 | -7.77 |
|  |  |  |  |  |  |  | P8-O1 | -6.69 |
|  |  |  |  |  |  |  | Fp1-O2 | -5.83 |
|  |  |  |  |  |  |  | Fp2-O2 | -5.77 |
|  |  |  |  |  |  |  | F7-O2 | -5.38 |
|  |  |  |  |  |  |  | C4-O2 | -5.23 |
|  |  |  |  |  |  |  | P4-O2 | -5.23 |
|  |  |  |  |  |  |  | P8-O2 | -8.03 |
|  |  |  |  |  |  |  | O1-O2 | -6.84 |
| SB  vs  PB | F4-F4 | 7.58 | F4-C3 | 5.30 | F7-T8 | -4.55 |  |  |
|  | F4-F8 | 6.20 | F4-C3 | 4.73 | C4-T8 | -4.76 |  |  |
|  | F7-C3 | 4.84 | P7-P3 | 4.68 | T8-P4 | -5.21 |  |  |
|  | F4-C3 | 5.38 | P3-P4 | -5.23 |  |  |  |  |
|  | F7-C4 | 6.44 |  |  |  |  |  |  |
|  | F4-C4 | 5.57 |  |  |  |  |  |  |
|  | C3-C4 | 4.68 |  |  |  |  |  |  |
|  | Fp1-T8 | 5.41 |  |  |  |  |  |  |
|  | F4-T8 | 7.97 |  |  |  |  |  |  |
|  | F4-T8 | 5.09 |  |  |  |  |  |  |
|  | T8-O1 | 4.92 |  |  |  |  |  |  |
